# Factors Associated with the Intention to Adopt Digital Health Technologies for Physical Activity Among People with Disabilities: An Integrated Technology Acceptance Model–Theory of Planned Behavior Framework

**DOI:** 10.3390/healthcare14101344

**Published:** 2026-05-14

**Authors:** Jinwoo Park, Hyunsu Lee, Yongchul Kwon, Gunsang Cho, Junghee Yun

**Affiliations:** Department of Physical Education, Pusan National University, Busan 46241, Republic of Korea; wave3305@pusan.ac.kr (J.P.); s3airlee@pusan.ac.kr (H.L.)

**Keywords:** digital health technology, physical activity, disabled people, technology acceptance model, theory of planned behavior, structural equation modeling, behavioral intention

## Abstract

**Highlights:**

**What are the main findings?**

- The integrated Technology Acceptance Model–Theory of Planned Behavior framework showed that attitude and subjective norm were the main positive correlates of intention to adopt digital health technologies for physical activity among people with disabilities.
- Perceived usefulness and perceived ease of use influenced intention mainly through indirect pathways, whereas perceived behavioral control showed an inverse association with intention.

**What are the implications of the main findings?**

- Adoption strategies should emphasize perceived benefits, positive user evaluations, accessible design, supportive encouragement from significant others, and implementation conditions that reduce structural barriers.
- Future research should distinguish self-efficacy from controllability within perceived behavioral control, examine actual use behavior, and compare findings across disability types and cultural contexts.

**Abstract:**

Background/Objectives: Digital health technologies may support physical activity participation among people with disabilities, yet their adoption remains limited because of accessibility, usability, and implementation barriers. This study examined factors associated with the intention to adopt digital health technologies for physical activity among people with disabilities using an integrated Technology Acceptance Model–Theory of Planned Behavior framework. Methods: Data were collected from 362 individuals with physical, visual, or hearing impairments who participated in community-based sports programs in South Korea. Structural equation modeling (SEM) was used to test the integrated model, and indirect effects were examined using bias-corrected bootstrapping. Results: The final model showed acceptable fit. Perceived ease of use was positively associated with perceived usefulness, and both perceived ease of use and perceived usefulness were positively associated with attitude and perceived behavioral control. Attitude and subjective norm were positively associated with intention to adopt digital health technologies, whereas perceived behavioral control showed a significant inverse association. Perceived usefulness and perceived ease of use were indirectly associated with intention primarily through attitude. Conclusions: The integrated framework provided a useful, although incomplete, explanation of digital health technology adoption intention among people with disabilities and suggests that perceived control may operate differently in disability-related digital health contexts. Adoption strategies should emphasize perceived benefits, positive user evaluations, accessible design, social support, and implementation conditions that reduce structural barriers to physical activity and health management.

## 1. Introduction

Digital health technologies—including mobile health applications, wearable activity trackers, telehealth platforms, and remote monitoring systems—have increasingly been used to support health management and promote physical activity. These technologies can facilitate self-monitoring, provide real-time feedback, and support more individualized health behaviors, thereby expanding opportunities for health promotion beyond conventional face-to-face settings.

For people with disabilities, regular participation in physical activity is especially important for maintaining functional capacity, preventing secondary health conditions, and enhancing quality of life. However, people with disabilities often experience lower levels of physical activity than the general population because of environmental and structural barriers such as limited accessibility, transportation difficulties, and restricted access to appropriate services and information. In this context, digital health technologies have been recognized as promising tools for reducing some of these barriers and supporting more accessible participation in physical activity. Recent reviews have shown that digital platforms and telehealth approaches may enhance access and flexibility for people with disabilities, although their implementation remains uneven and evidence for sustained adoption is still limited [1,2]. In addition, research on digital interventions for promoting physical activity among people with intellectual disabilities and/or autism suggests potential benefits, while also highlighting substantial variation in intervention quality, user engagement, and methodological rigor [3]. Studies of remote disability services have likewise emphasized that usability, accessibility, and service support are central to successful technology use [4].

Despite this potential, the adoption and sustained use of digital health technologies among people with disabilities remain limited. Individuals in this population may encounter challenges related to usability, accessibility, cost, digital literacy, and implementation support, all of which can constrain effective engagement with digital health tools [1,2,4]. These findings suggest that the value of digital health technologies depends not only on their technical capabilities but also on how users perceive their usefulness, usability, social support, and controllability.

To explain such adoption processes, researchers have frequently used the Technology Acceptance Model (TAM) and the Theory of Planned Behavior (TPB). TAM emphasizes perceived usefulness and perceived ease of use as core determinants of technology-related attitudes and behavioral intention [5], and previous digital health adoption studies have shown that these technology-related beliefs are important for understanding users’ acceptance of health technologies [6,7,8]. Although both frameworks have been widely applied in health behavior and technology adoption research, relatively few studies have integrated TAM and TPB to explain the intention to adopt digital health technologies for physical activity among people with disabilities. Recent disability-focused and digital health implementation studies further suggest that adoption is shaped not only by perceived usefulness and ease of use but also by accessibility, service support, contextual fit, and implementation conditions [1,2,4]. An integrated framework may therefore provide a more comprehensive explanation by capturing both technology-specific beliefs and broader social–cognitive determinants of behavioral intention, particularly in disability-related contexts where technology adoption is closely tied to usability, support structures, and environmental accessibility [1,2,4].

Accordingly, the purpose of this study was to examine the factors associated with the intention to adopt digital health technologies for physical activity among people with disabilities. Specifically, this study integrated TAM and TPB to investigate how perceived ease of use, perceived usefulness, attitude, subjective norm, and perceived behavioral control are associated with intention to adopt digital health technologies. By identifying these correlates, this study aims to provide empirical evidence to inform the design of accessible digital health interventions and policy strategies that promote physical activity participation among people with disabilities.

This study contributes to the literature in four ways. First, it extends digital health technology acceptance research by applying an integrated Technology Acceptance Model–Theory of Planned Behavior framework to the context of physical activity among people with disabilities. Second, it focuses on individuals with physical, visual, and hearing impairments who participate in community-based sports programs, thereby providing evidence from a population that remains underrepresented in digital health adoption research. Third, it compares a full structural model with a more parsimonious model and examines indirect pathways, clarifying whether perceived usefulness and perceived ease of use are directly or indirectly associated with adoption intention. Fourth, it highlights the complex role of perceived behavioral control in disability-related digital health contexts, particularly the need to distinguish self-efficacy from controllability.

The remainder of this paper is organized as follows. Section 2 presents the theoretical background and hypotheses. Section 3 describes the participants, measures, data collection procedures, and analytical strategy. Section 4 reports the measurement model, structural model, model comparison, and indirect effect results. Section 5 discusses the findings, health implications, limitations, and future research directions. Section 6 concludes the study.

## 2. Theoretical Background and Hypotheses Development

### 2.1. Technology Acceptance Model and Digital Health Technology Use

The Technology Acceptance Model (TAM) explains technology adoption through two core beliefs: perceived usefulness and perceived ease of use [5]. Perceived usefulness refers to the extent to which an individual believes that using a particular technology will enhance their performance or outcomes, whereas perceived ease of use refers to the degree to which an individual believes that using the technology will require minimal effort. These perceptions are theorized to influence users’ attitudes toward technology use and subsequently shape their behavioral intentions.

In the field of digital health, TAM has been extensively applied to examine the adoption of health-related technologies, including mobile health applications, wearable activity trackers, telehealth services, and remote health monitoring systems. Previous studies have consistently reported that perceived usefulness and perceived ease of use are associated with individuals’ acceptance of digital health technologies [6,7,8]. When users perceive a digital health technology as beneficial for managing health and easy to operate, they are more likely to adopt the technology and integrate it into their daily health management behaviors.

A fundamental assumption of TAM is that perceived ease of use is related to perceived usefulness. Technologies that are easier to operate reduce the effort required to perform tasks, which may lead users to perceive them as more beneficial and efficient. This relationship has been consistently supported in previous technology acceptance research [5,9]. In digital health environments, user-friendly interfaces, intuitive system design, and simplified interaction processes may enhance users’ perceptions of usefulness, particularly among populations who may experience technological barriers.

Perceived usefulness and perceived ease of use are also considered important determinants of attitudes toward technology use. Individuals who believe that digital health technologies are beneficial for improving health management and are easy to use are more likely to develop positive attitudes toward their use. These positive attitudes may subsequently strengthen individuals’ intention to adopt such technologies.

Furthermore, the perceived ease of use and perceived usefulness of digital health technologies may enhance individuals’ perceived behavioral control regarding technology use. When digital health technologies are perceived as simple and easy to operate, individuals may feel more confident in their ability to use these technologies effectively. Similarly, when technologies are perceived as useful, individuals may feel more capable of integrating them into their daily routines. In other words, technologies that are perceived as easy to use and useful may increase individuals’ perceptions that they possess the necessary skills and resources to utilize them.

In addition to shaping attitudes and perceived behavioral control, perceived usefulness and perceived ease of use may directly relate to individuals’ behavioral intention to use digital health technologies. Several studies in extended TAM frameworks have reported direct associations between these constructs and intention [6,9]. When individuals perceive digital health technologies as useful for managing their health and easy to operate, they may be more willing to adopt and utilize these technologies in their daily lives.

Based on these theoretical perspectives, the following hypotheses are proposed:

**H1:**  *Perceived ease of use will be positively associated with perceived usefulness of digital health technologies.*

**H2:**  *Perceived ease of use will be positively associated with attitudes toward the use of digital health technologies.*

**H3:**  *Perceived usefulness will be positively associated with attitudes toward the use of digital health technologies.*

**H4:**  *Perceived ease of use will be positively associated with perceived behavioral control regarding the use of digital health technologies.*

**H5:**  *Perceived usefulness will be positively associated with perceived behavioral control regarding the use of digital health technologies.*

**H6:**  *Perceived usefulness will be positively associated with the intention to adopt digital health technologies.*

**H7:**  *Perceived ease of use will be positively associated with the intention to adopt digital health technologies.*

### 2.2. Theory of Planned Behavior and Technology Use Intention

The Theory of Planned Behavior (TPB) explains behavioral intention through three key determinants: attitude, subjective norm, and perceived behavioral control [10]. Attitude refers to an individual’s overall positive or negative evaluation of performing a particular behavior. Subjective norm represents the perceived social pressure from significant others, such as family members, friends, peers, or professionals, regarding whether the individual should perform the behavior. Perceived behavioral control (PBC) reflects an individual’s perception of the ease or difficulty of performing the behavior and is associated with beliefs about the availability of resources, skills, and opportunities necessary to perform that behavior.

TPB has been widely applied in research examining health-related behaviors, including physical activity participation, health promotion behaviors, and the adoption of health technologies. Previous studies have consistently demonstrated that individuals who hold positive attitudes toward a behavior are more likely to develop stronger intentions to engage in that behavior. In the context of digital health technologies, individuals who evaluate the use of such technologies positively are more likely to intend to adopt and utilize them as part of their health management and physical activity practices.

Subjective norm also plays an important role in shaping behavioral intention. Individuals may be influenced by the expectations or encouragement of significant others, including family members, peers, coaches, or healthcare professionals. When individuals perceive that important people in their social environment support or recommend the use of digital health technologies, they may be more motivated to adopt those technologies. For people with disabilities, who often rely on social networks for health information and support [11], social influence may be particularly salient in shaping technology adoption decisions.

Perceived behavioral control is another key determinant of behavioral intention. Individuals who believe that they possess sufficient knowledge, skills, and resources to perform a behavior are more likely to develop stronger intentions to engage in that behavior. In the context of digital health technologies, individuals who feel confident in their ability to operate digital devices, access technological resources, and manage digital tools may be more likely to adopt these technologies for health management and physical activity participation.

It is noteworthy that Ajzen [12] later clarified that perceived behavioral control comprises two conceptually distinct sub-dimensions: self-efficacy and controllability. Self-efficacy refers to perceived personal capability to perform the behavior, whereas controllability refers to beliefs about the extent to which performing the behavior is under one’s volitional control—that is, the perceived influence of external factors such as resources, opportunities, and environmental conditions. Terry and O’Leary [13] empirically demonstrated that these two dimensions can exert divergent effects on behavioral intention, and Rhodes and Courneya [14] further showed that measurement of PBC using controllability-oriented items can yield substantially different predictive patterns than self-efficacy-oriented items. This distinction may be particularly relevant for people with disabilities, who may experience a dissociation between their perceived personal capability (self-efficacy) and the perceived external constraints imposed by inaccessible technology design, cost barriers, and inadequate institutional support (controllability). The implications of this dual-dimensional structure are further elaborated in Section 5.

Based on the theoretical assumptions of TPB, the following hypotheses are proposed:

**H8:**  *Attitude toward the use of digital health technologies will be positively associated with the intention to adopt digital health technologies.*

**H9:**  *Subjective norm will be positively associated with the intention to adopt digital health technologies.*

**H10:**  *Perceived behavioral control will be positively associated with the intention to adopt digital health technologies.*

### 2.3. Integrated TAM–TPB Framework

Although the Technology Acceptance Model (TAM) provides a useful framework for explaining individuals’ perceptions of technology, it primarily focuses on technological beliefs such as perceived usefulness and perceived ease of use. As a result, TAM does not fully capture the broader psychological and social factors that may be associated with technology adoption. In contrast, the Theory of Planned Behavior explains behavioral intention from a broader perspective by incorporating social influence and perceived control over behavior.

Integrating TAM and TPB can therefore provide a more comprehensive framework for understanding technology adoption behavior. In the context of physical activity participation among people with disabilities, individuals’ perceptions of the usefulness and ease of use of digital health technologies may shape their attitudes toward technology use and their perceived ability to use such technologies. These perceptions, together with social influences from significant others, may ultimately be associated with individuals’ intention to use digital health technologies.

Accordingly, this study integrates TAM and TPB to examine the correlates of digital health technology use intention in the context of physical activity among people with disabilities. In the proposed research model, perceived ease of use is hypothesized to be associated with perceived usefulness (H1), attitudes toward technology use (H2), and perceived behavioral control (H4). Perceived usefulness is hypothesized to be associated with attitudes (H3), perceived behavioral control (H5), and intention to use (H6). Perceived ease of use may also be directly associated with intention to use (H7). Based on TPB, attitude (H8), subjective norm (H9), and perceived behavioral control (H10) are proposed as key correlates of behavioral intention.

**Figure 1:**
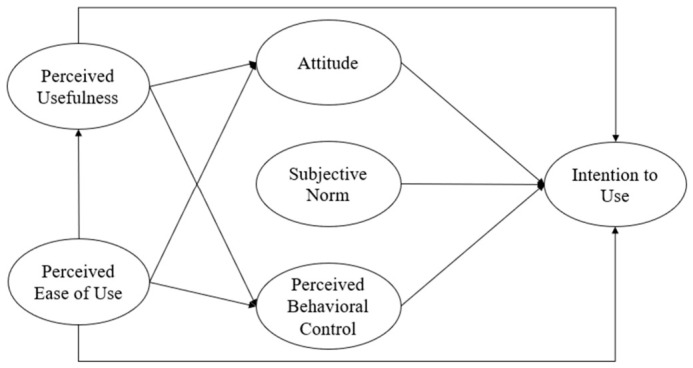
Hypothesized Integrated Technology Acceptance Model–Theory of Planned Behavior Framework.

Figure 1 presents the hypothesized integrated Technology Acceptance Model–Theory of Planned Behavior framework tested in this study.

The model was not designed as two separate parallel analyses of TAM and TPB. Rather, TAM-based variables and TPB-based variables were integrated into a single structural equation model. In this model, perceived ease of use and perceived usefulness represent technology-specific beliefs, whereas attitude, subjective norm, and perceived behavioral control represent broader social–cognitive determinants of behavioral intention. The hypothesized model was first evaluated through confirmatory factor analysis to verify the measurement structure and then tested through structural equation modeling. After the full model was evaluated, a more parsimonious final model was derived by removing non-significant direct paths and comparing model fit. Thus, Figure 1 represents the initial hypothesized model, whereas the final structural model supported by the data is presented later in Section 4.

Figure 1 visually summarizes the theoretical logic of the integrated model. The left side of the model represents TAM-based technology beliefs, including perceived ease of use and perceived usefulness, whereas the right side incorporates TPB-based determinants, including attitude, subjective norm, perceived behavioral control, and intention. This structure illustrates how technology-specific evaluations may operate together with social and control-related beliefs to explain adoption intention in the context of digital health technologies for physical activity.

## 3. Methods

### 3.1. Participants

Participants in this study were individuals with disabilities who were actively involved in community-based sports or physical activity programs across South Korea. A convenience sampling method was used to recruit participants from adaptive sports clubs and community-based physical activity settings, and data were collected in late March 2026.

Eligibility was limited to individuals with disabilities who were currently participating in regular physical activity or sports. Individuals with physical, visual, or hearing impairments were eligible to participate. However, individuals with developmental disabilities were excluded because substantial difficulty in understanding and independently completing the questionnaire, even with additional explanation, could compromise response validity.

A total of 400 questionnaires were distributed, and 362 valid responses were retained after excluding incomplete responses, missing data, and systematic response patterns. The final sample included individuals with physical, visual, and hearing impairments who were participating in community-based sports or physical activity programs. Demographic, disability-related, and digital health technology experience characteristics of the participants are summarized in Table 1.

### 3.2. Data Collection

Data were collected using an offline questionnaire survey. The research team visited sports facilities and community-based physical activity program sites where individuals with disabilities regularly participated. After providing a brief explanation of the study purpose and procedures, questionnaires were distributed to participants who agreed to participate. To promote consistent administration and reduce potential response bias, all research assistants followed standardized survey procedures and received prior training before the data collection period. Participants were given clear instructions on how to complete the questionnaire, and assistance was provided when necessary because of physical or sensory limitations. Such assistance was limited to facilitating access to the survey process, such as reading items aloud or recording verbal responses, and did not involve interpreting questionnaire items on behalf of participants. The questionnaire consisted of items assessing the six constructs included in the integrated TAM–TPB model, along with demographic questions. All items were presented in Korean, and the wording was reviewed in advance to enhance clarity and comprehensibility for participants with disabilities. Participants completed the questionnaire anonymously, and no personally identifiable information was collected.

### 3.3. Measures

All constructs were assessed using previously validated instruments adapted for the context of digital health technology use for physical activity. Item wording was modified to reflect the use of digital health technologies, including wearable devices, mobile health applications, and online exercise platforms, in relation to physical activity participation among people with disabilities.

Perceived ease of use (PEOU) was assessed with four items measuring the extent to which participants perceived digital health technologies as easy to use and free of effort. These items were adapted from Davis [5] and subsequent digital health studies [8,9].

Perceived usefulness (PU) was assessed with four items measuring the extent to which participants perceived digital health technologies as beneficial for health management and physical activity participation. These items were adapted from Davis [5] and prior digital health adoption studies [6,7].

Attitude (ATT) was assessed with five items measuring participants’ overall evaluation of using digital health technologies for physical activity. These items were adapted from the Theory of Planned Behavior literature [10] and related technology adoption studies.

Subjective norm (SN) was assessed with three items measuring perceived social pressure from significant others, including family members, friends, healthcare professionals, and coaches, regarding the use of digital health technologies. These items were adapted from Ajzen [10].

Perceived behavioral control (PBC) was assessed with three items reflecting a mixed sense of personal capability and situational control over the use of digital health technologies for physical activity. These items were adapted from Ajzen [10] and health behavior research. Two items primarily reflected self-efficacy-oriented beliefs, whereas one item also reflected circumstance-related controllability. This mixed operationalization has implications for the interpretation of results, as discussed in Section 5.

Intention to use (INT) was assessed with four items measuring participants’ intention to adopt and use digital health technologies for physical activity and health management. These items were adapted from the technology acceptance and health behavior literature.

All items were rated on a five-point Likert scale ranging from 1 (strongly disagree) to 5 (strongly agree). The final measurement items are presented in Table 2.

### 3.4. Data Analysis

Data analysis was conducted using IBM SPSS Statistics 28.0 (IBM Corp., Armonk, NY, USA, 2021) and IBM SPSS Amos 28.0 (IBM Corp., Armonk, NY, USA, 2021) in several sequential steps. First, descriptive statistics were calculated to summarize participant characteristics and the central tendencies of the study variables. Second, confirmatory factor analysis (CFA) was conducted to evaluate whether the observed items adequately represented the six latent constructs included in the hypothesized model shown in Figure 1. Third, internal consistency reliability and construct validity were examined using Cronbach’s alpha, composite reliability (CR), average variance extracted (AVE), the Fornell–Larcker criterion, and the heterotrait–monotrait ratio (HTMT). Fourth, the full hypothesized structural model was tested using structural equation modeling (SEM). Fifth, a parsimonious model was evaluated by removing the non-significant direct paths from perceived usefulness and perceived ease of use to intention, and the full and parsimonious models were compared using fit indices, the Akaike information criterion (AIC), and chi-square difference testing. Sixth, indirect effects were examined using bias-corrected bootstrapping with 5000 resamples. Seventh, additional diagnostic analyses were conducted to assess common method variance and the possibility of suppression or multicollinearity in the relationship between perceived behavioral control and intention.

## 4. Results

### 4.1. Measurement Model: Confirmatory Factor Analysis

The results are presented in four subsections to reflect the analytical sequence of the study. First, the measurement model is evaluated through confirmatory factor analysis. Second, discriminant validity is examined using the Fornell–Larcker criterion and HTMT ratios. Third, the hypothesized and parsimonious structural models are compared, and the final hypothesis testing results are reported. Finally, indirect effects are examined using bias-corrected bootstrapping. This organization follows the logic of first establishing the adequacy of the measurement model and then interpreting the structural relationships among the latent constructs.

Confirmatory factor analysis (CFA) was conducted to evaluate the measurement model. The results indicated an acceptable overall model fit (χ^2^ = 513.240, df = 215, χ^2^/df = 2.387, CFI = 0.939, TLI = 0.929, NFI = 0.901, RMSEA = 0.062, 90% CI [0.055, 0.069]). The measurement model showed acceptable fit according to the criteria of χ^2^/df < 3.00, CFI and TLI ≥ 0.90, and RMSEA ≤ 0.08 [15,16].

The standardized factor loadings for all items are presented in Table 3. All factor loadings were statistically significant (*p* < 0.001) and ranged from 0.507 to 0.936. Composite reliability (CR) values ranged from 0.749 to 0.900, all exceeding the recommended threshold of 0.70. Average variance extracted (AVE) values ranged from 0.510 to 0.698, and all constructs met the recommended criterion of 0.50 or above [17]. Taken together, these results provide support for convergent validity.

### 4.2. Discriminant Validity

Discriminant validity was assessed using the Fornell–Larcker criterion, whereby the square root of the average variance extracted (AVE) for each construct should exceed its correlations with other constructs. As shown in Table 4, the square root of the AVE for most constructs exceeded the corresponding inter-construct correlations, providing general support for discriminant validity.

One exception was observed for the relationship between attitude and perceived usefulness. The correlation between these two constructs (0.792) slightly exceeded the square root of the AVE for attitude (0.767), suggesting a modest degree of conceptual overlap. To further examine discriminant validity, heterotrait-monotrait (HTMT) ratios were calculated. All HTMT values ranged from 0.169 to 0.812 and were below the commonly used threshold of 0.85. Specifically, the HTMT ratio between perceived usefulness and attitude was 0.812, supporting the empirical distinction between these constructs despite their high correlation. The theoretical distinction between technology-related instrumental beliefs and evaluative attitude also supports retaining these constructs as separate factors within the integrated framework.

### 4.3. Structural Model and Hypothesis Testing

Following confirmation of an acceptable measurement model, two structural models were compared to test the hypothesized relationships. Model 1 (full model) included all hypothesized paths, including the direct paths from perceived usefulness (PU) and perceived ease of use (PEOU) to intention (INT), whereas Model 2 (parsimonious model) excluded these two non-significant direct paths. The comparison between the two models is presented in Table 5.

**Figure 2:**
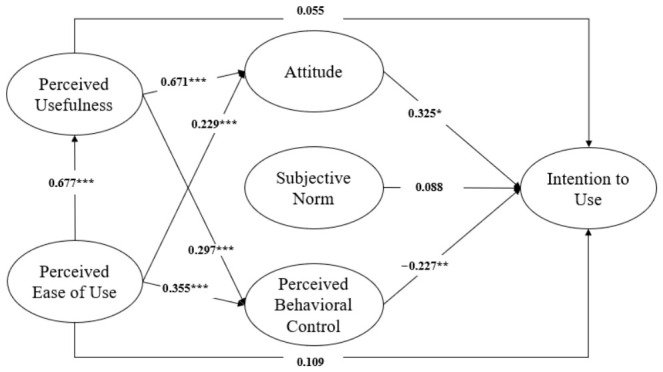
Structural model results for Model 1 (full hypothesized model). Standardized coefficients are presented; * *p* < 0.05, ** *p* < 0.01, *** *p* < 0.001.

Figure 2 presents the full hypothesized structural model before model trimming. This figure is included to show the complete set of theoretically proposed paths and to clarify why the direct paths from perceived usefulness and perceived ease of use to intention were not retained in the final model. As shown in the full model, these two direct paths were not statistically significant, whereas the indirect pathways through attitude remained theoretically and empirically meaningful.

**Figure 3:**
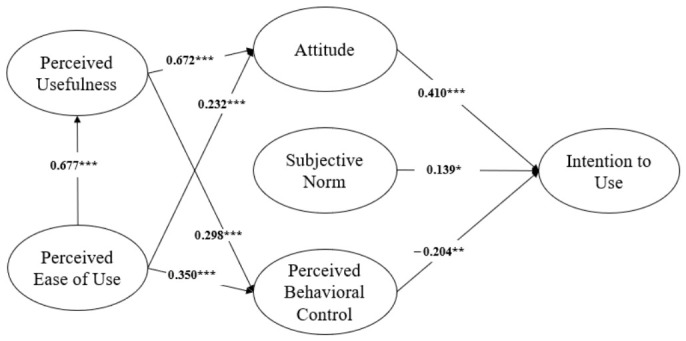
Structural model results for Model 2 (final parsimonious model). Standardized coefficients are presented; * *p* < 0.05, ** *p* < 0.01, *** *p* < 0.001.

As shown in Table 5, Model 1 and Model 2 demonstrated very similar levels of overall model fit. For Model 1, the fit indices were χ^2^/df = 2.545, CFI = 0.931, TLI = 0.920, and RMSEA = 0.065. For Model 2, the corresponding values were χ^2^/df = 2.528, CFI = 0.931, TLI = 0.921, and RMSEA = 0.065. In addition, Model 2 yielded a lower AIC value (668.247) than Model 1 (670.705), indicating better model parsimony. Because Model 1 and Model 2 were nested, a chi-square difference test was conducted to examine whether removing the two non-significant direct paths significantly worsened model fit. The difference was not statistically significant (Δχ^2^ = 1.542, Δdf = 2, *p* = 0.463), indicating that the more parsimonious model did not fit significantly worse than the full model. In Model 1, the direct path from PU to intention was not statistically significant (β = 0.055, *p* = 0.662), and the direct path from PEOU to intention was also not statistically significant (β = 0.109, *p* = 0.353). Accordingly, these two paths were excluded from Model 2, which was retained as the final structural model. The overall fit of the final model (Model 2) was acceptable (χ^2^ = 556.247, df = 220, χ^2^/df = 2.528, CFI = 0.931, TLI = 0.921, RMSEA = 0.065, 90% CI [0.058, 0.072]). The final hypothesis testing results for Model 2 are summarized in Table 6. Figure 3 presents the final model after the non-significant direct paths from perceived usefulness and perceived ease of use to intention were removed.

With respect to the TAM-based hypotheses, H1 predicted that perceived ease of use (PEOU) would be positively associated with perceived usefulness (PU). This hypothesis was supported (β = 0.677, *p* < 0.001), indicating that individuals who perceived digital health technologies as easy to use were more likely to perceive them as useful for health management. H2 and H3 examined the associations of PEOU and PU with attitude, respectively, and both were supported (H2: β = 0.232, *p* < 0.001; H3: β = 0.672, *p* < 0.001). H4 and H5 proposed that PEOU and PU would be positively associated with perceived behavioral control (PBC), and both hypotheses were supported (H4: β = 0.350, *p* < 0.001; H5: β = 0.298, *p* < 0.001). As shown in Table 5, H6 and H7, which predicted direct associations of PU and PEOU with intention, were not supported in Model 1 and were therefore excluded from the final model.

Regarding the TPB-based hypotheses, H8 predicted that attitude would be positively associated with intention to use, and this hypothesis was strongly supported (β = 0.410, *p* < 0.001). H9 proposed that subjective norm would be positively associated with intention, and this hypothesis was supported in the final model (β = 0.139, *p* = 0.042). H10 predicted that perceived behavioral control would be positively associated with intention; however, this hypothesis was not supported in the expected direction. Instead, perceived behavioral control showed a statistically significant negative association with intention (β = −0.204, *p* = 0.008). The final structural model explained 18.1% of the variance in intention to use (R^2^ = 0.181).

Additional diagnostic analyses were conducted to address potential common method variance and suppression. Harman’s single-factor test indicated that the first unrotated factor accounted for 38.908% of the total variance, which was below the commonly used 50% threshold. This result suggests that common method variance was unlikely to be a dominant source of bias. Variance inflation factors (VIFs) were also calculated for the three direct predictors of intention in the final model. The VIF values were 2.310 for attitude, 2.163 for subjective norm, and 1.674 for perceived behavioral control, indicating that severe multicollinearity was unlikely. Supplementary regression diagnostics using the construct correlation matrix further showed that perceived behavioral control had a near-zero bivariate correlation with intention (r = 0.052) but showed a negative standardized coefficient when attitude, subjective norm, and perceived behavioral control were entered simultaneously as predictors of intention (β = −0.284). This pattern is consistent with a suppression effect rather than a simple negative bivariate association.

### 4.4. Indirect Effects: Bootstrapping Analysis

Bias-corrected (BC) bootstrapping with 5000 resamples was conducted to examine the indirect effects in the final structural model (Model 2). The results are presented in Table 7. For inferential testing, statistical significance was determined using unstandardized bias-corrected confidence intervals, whereas standardized indirect effects are reported to facilitate interpretation of effect size.

For perceived ease of use (PEOU), the standardized total indirect effect on intention to use was β = 0.169. The unstandardized bias-corrected bootstrap test indicated that this indirect effect was statistically significant (BC *p* = 0.004, 95% CI [0.042, 0.284]), as the confidence interval did not include zero. Decomposition of this total indirect effect identified four constituent pathways: a positive indirect path through attitude (PEOU → ATT → INT, β = 0.095), a positive sequential path through perceived usefulness and attitude (PEOU → PU → ATT → INT, β = 0.186), a negative indirect path through perceived behavioral control (PEOU → PBC → INT, β = −0.071), and an additional negative sequential path through perceived usefulness and perceived behavioral control (PEOU → PU → PBC → INT, β = −0.041). Taken together, these results indicate that the positive pathways through attitude outweighed the negative pathways through perceived behavioral control, resulting in a net positive total indirect effect.

For perceived usefulness (PU), the standardized total indirect effect on intention was β = 0.214. The unstandardized bias-corrected bootstrap test likewise indicated that this indirect effect was statistically significant (BC *p* < 0.001, 95% CI [0.068, 0.207]), as the confidence interval did not include zero. Two indirect pathways were identified: a positive path through attitude (PU → ATT → INT, β = 0.276) and a negative path through perceived behavioral control (PU → PBC → INT, β = −0.061). Because the positive indirect path through attitude was stronger than the negative path through perceived behavioral control, the overall indirect effect of PU on intention remained positive.

Overall, these findings indicate that attitude served as the primary positive mediating mechanism through which both PEOU and PU were associated with the intention to use digital health technologies, whereas perceived behavioral control functioned as a negative pathway in this population.

## 5. Discussion

The present study examined the correlates of intention to adopt digital health technologies for physical activity among people with disabilities using an integrated Technology Acceptance Model–Theory of Planned Behavior framework. Overall, the findings largely supported the proposed model. Perceived ease of use and perceived usefulness were positively associated with attitude and perceived behavioral control, attitude and subjective norm were positively associated with intention, and perceived behavioral control showed an unexpected negative association with intention. In addition, perceived ease of use and perceived usefulness were associated with intention primarily through indirect rather than direct pathways.

The final model explained 18.1% of the variance in intention, indicating that the integrated framework provided a meaningful but incomplete explanation of digital health technology adoption in this population [18]. This modest explanatory power suggests that intention among people with disabilities may be shaped by additional factors beyond technology-related beliefs and general social–cognitive variables. In disability-related digital health contexts, perceived accessibility, digital literacy, affordability, privacy concerns, trust in technology, prior technology experience, availability of technical assistance, instructor or caregiver support, and disability-specific usability needs may be particularly important. Future research should therefore extend the integrated framework by incorporating these contextual and disability-specific variables. Qualitative or mixed-method approaches may also be useful for identifying determinants of adoption intention that are not fully captured by standardized acceptance models. To further contextualize the present findings, Table 8 compares the integrated framework used in this study with recent digital health technology acceptance and implementation studies.

As shown in Table 8, previous studies have primarily focused on general digital health acceptance, telehealth implementation, digital intervention sustainability, or digital health technology evaluation. The present study extends this literature by integrating technology-specific beliefs and social–cognitive determinants in the context of physical activity among people with disabilities.

### 5.1. TAM-Based Findings

Consistent with TAM, perceived ease of use was positively associated with perceived usefulness, indicating that participants who perceived digital health technologies as easier to use were more likely to regard them as functionally beneficial. This finding accords with prior digital health technology research showing that ease of use remains an important antecedent of perceived usefulness [5,6,8]. In disability-related contexts, this result is especially meaningful because recent reviews have emphasized that usability, accessibility, and implementation support are central to meaningful digital engagement [1,2,4].

Both perceived ease of use and perceived usefulness were positively associated with attitude, but perceived usefulness showed a substantially stronger association. This suggests that perceived functional value, rather than operational simplicity alone, was more strongly related to favorable evaluations of digital health technologies. This interpretation is consistent with recent digital health adoption research indicating that perceived benefit is one of the strongest correlates of technology acceptance [7]. It is also broadly consistent with recent disability-focused reviews showing that digital interventions are more likely to be accepted when users perceive clear practical benefits for health management, participation, and daily functioning [2,19].

Both perceived ease of use and perceived usefulness were also positively associated with perceived behavioral control, suggesting that technologies viewed as usable and beneficial may strengthen users’ sense of capability in using them. However, neither construct was directly associated with intention in Model 1. Instead, both constructs were associated with intention indirectly in the final model. For perceived ease of use, the strongest positive pathway operated through perceived usefulness and attitude, whereas negative indirect pathways operated through perceived behavioral control. For perceived usefulness, the positive indirect pathway through attitude outweighed the negative pathway through perceived behavioral control. Taken together, these findings suggest that TAM constructs were associated with intention primarily by shaping positive evaluations rather than by directly motivating adoption. This pattern is consistent with TAM-based theory in which ease of use often exerts its influence through usefulness and attitude rather than through a direct path to intention [5,9].

### 5.2. TPB-Based Findings

In line with TPB, attitude was positively associated with intention to use digital health technologies. This indicates that favorable evaluations of digital health technology use are closely linked to stronger adoption intention among people with disabilities. The finding is consistent with broader health behavior research identifying attitude as a robust correlate of intention [18], and it aligns with recent disability-related digital health research suggesting that perceived value and relevance are important for sustained engagement [2,3].

Subjective norm was also positively associated with intention in the final model. This finding suggests that support or encouragement from significant others, such as family members, peers, instructors, or healthcare professionals, may play an important role in technology adoption decisions among people with disabilities. Recent studies on telehealth and remote disability services similarly indicate that social and service support are often critical to successful uptake, particularly when users need assistance in navigating systems, accessing information, or sustaining engagement over time [4,19].

### 5.3. Interpreting the Negative Association Between Perceived Behavioral Control and Intention

Contrary to H10, perceived behavioral control showed a significant negative association with intention. This finding should be interpreted cautiously and should not be taken as evidence that greater perceived control necessarily reduces adoption intention among people with disabilities. One plausible explanation is that the present PBC measure combined self-efficacy-oriented items with one item reflecting circumstance-related controllability. Accordingly, the negative coefficient may reflect conceptual heterogeneity within the unidimensional operationalization of PBC rather than a direct substantive effect of control beliefs themselves. This interpretation is consistent with Ajzen’s [12] clarification that perceived behavioral control comprises conceptually distinct elements, and with prior work suggesting that self-efficacy and controllability may show different relationships with intention [13,14].

The negative structural coefficient for perceived behavioral control may also reflect a suppression pattern. In the present data, the bivariate correlation between perceived behavioral control and intention was close to zero, whereas the structural coefficient became negative after attitude and subjective norm were included in the model. This pattern suggests that perceived behavioral control may share positive variance with other predictors, particularly attitude, while its residual component may capture perceived external constraints or conditional controllability. Therefore, the negative coefficient should not be interpreted as evidence that greater perceived control reduces adoption intention. Rather, it indicates that perceived behavioral control may operate heterogeneously in disability-related digital health contexts, especially when self-efficacy and controllability are combined within a single measure. Additional multicollinearity diagnostics indicated that the VIF values for attitude, subjective norm, and perceived behavioral control ranged from 1.674 to 2.310, suggesting that severe multicollinearity was unlikely. Supplementary regression diagnostics also showed that perceived behavioral control had a near-zero bivariate correlation with intention (r = 0.052) but became negative when attitude and subjective norm were included as simultaneous predictors of intention (β = −0.284).

This interpretation may be particularly relevant in disability-related digital health contexts. People with disabilities may perceive themselves as personally capable of using a technology while simultaneously recognizing substantial external barriers, such as inaccessible design, insufficient support, cost, or implementation constraints. Recent reviews of telehealth and disability services have consistently identified accessibility, training, service support, and system design as major determinants of digital engagement [2,4,19]. Within such contexts, stronger perceived capability may not always translate into stronger intention if anticipated structural barriers remain salient. Thus, the negative association observed in the present model may reflect the mixed operationalization of perceived control in a context where personal capability and environmental controllability do not necessarily align.

Therefore, the present finding should not be interpreted as a direct contradiction of TPB. Rather, it suggests that the role of perceived behavioral control in disability-related digital health contexts may require greater conceptual specificity than is typically assumed in a unidimensional model. Future research should measure self-efficacy and controllability separately and should directly assess related factors such as accessibility barriers, digital literacy, prior technology experience, and support availability.

### 5.4. Practical Implications

The present findings yield several practical implications. First, because perceived usefulness showed the strongest association with attitude, developers and health practitioners should clearly communicate the concrete benefits of digital health technologies for health management and physical activity participation. This implication is consistent with recent work on early-stage assessment of digital health technologies, which emphasizes that perceived value, implementation feasibility, end-user relevance, and contextual fit should be considered before large-scale deployment [22]. Second, the significant role of subjective norm suggests that adoption may be strengthened through supportive social environments involving family members, peers, instructors, and healthcare professionals. Third, the associations of perceived ease of use with both usefulness and perceived behavioral control highlight the importance of accessible and user-friendly design. Recent disability-focused reviews likewise emphasize that accessibility, usability, and support structures are central to equitable digital health implementation [1,2,19], and broader digital health implementation research similarly highlights the importance of end-user support, organizational readiness, and implementation processes [23]. Finally, the negative PBC finding suggests that efforts to promote adoption should not focus only on individual capability, but also on reducing structural barriers such as cost, accessibility limitations, and lack of technical support.

From a health promotion perspective, digital health technology adoption among people with disabilities should be understood not merely as a matter of technology use but also as a pathway to supporting physical activity participation, self-management, and long-term health equity. Accessible digital tools may help users monitor activity, receive feedback, sustain motivation, and overcome barriers related to transportation, service availability, and limited access to specialized physical activity programs. Because regular physical activity is important for maintaining functional capacity and preventing secondary health conditions, improving the acceptability and accessibility of digital health technologies may contribute to broader health promotion strategies for people with disabilities.

### 5.5. Limitations and Future Research

Several limitations warrant consideration. First, the cross-sectional design precludes causal inference. Second, participants were recruited from community-based sports programs in South Korea, and individuals with developmental disabilities were excluded. Therefore, the findings may not generalize to people with disabilities who are not physically active, those who face more severe participation barriers, individuals with developmental disabilities, or people with disabilities in other cultural and healthcare contexts. Cross-disability and cross-cultural comparisons are needed to examine whether the integrated framework operates similarly across disability types, service systems, and cultural environments. Third, all variables were assessed through self-report questionnaires at a single time point. Although Harman’s single-factor test suggested that common method variance was unlikely to be a dominant source of bias, common method bias cannot be fully ruled out. Fourth, disability type was not analyzed as a moderating factor, despite likely differences in accessibility needs and technology use conditions. The sample was also weighted toward participants with physical impairments, which may further limit the generalizability of the findings across disability types. Fifth, although the sample size exceeded commonly recommended minimums for SEM, a formal a priori power analysis was not conducted. Subsequent studies should conduct a priori power analysis based on anticipated model complexity, expected effect size, and desired statistical power.

Moreover, the relatively modest explanatory power of the model (R^2^ = 0.181) suggests that digital health technology adoption among people with disabilities is shaped by factors beyond those included in the present framework. The mixed operationalization of perceived behavioral control should also be regarded as a methodological limitation; therefore, subsequent studies should assess self-efficacy and controllability as distinct constructs. Expanded models should incorporate variables such as perceived accessibility, digital literacy, affordability, prior technology experience, privacy concerns, trust in technology, disability-specific needs, and social or institutional support, as broader digital intervention research increasingly emphasizes contextual fit, implementation readiness, user engagement, and long-term sustainability [21]. In addition, longitudinal, qualitative, or mixed-method designs would be useful for examining actual use behavior, comparing specific categories of digital health technologies, and identifying adoption determinants that may not be fully captured by standardized acceptance models. Finally, research on wearable and sensor-based systems should address long-term technical reliability because sustained physical activity monitoring may be affected by environmental variation, sensor accuracy, device stability, and system robustness [20].

## 6. Conclusions

This study investigated factors associated with the intention to adopt digital health technologies for physical activity among people with disabilities using an integrated Technology Acceptance Model–Theory of Planned Behavior framework. Based on structural equation modeling of data from 362 individuals with physical, visual, or hearing impairments participating in community-based sports programs in South Korea, the findings generally supported the proposed model and explained 18.1% of the variance in adoption intention.

Consistent with TAM, perceived ease of use was positively associated with perceived usefulness, and both constructs were positively associated with attitude and perceived behavioral control. Perceived usefulness showed a stronger association with attitude than perceived ease of use, suggesting that perceived functional value was more strongly related to favorable evaluations of digital health technologies. In addition, neither perceived usefulness nor perceived ease of use was directly associated with intention in the full model; instead, both constructs were associated with intention primarily through indirect pathways. In this process, attitude served as the primary positive mediating pathway, whereas perceived behavioral control functioned as a negative pathway.

Within the TPB framework, attitude and subjective norm were positively associated with intention to use digital health technologies. However, perceived behavioral control showed a significant negative association with intention. This finding should be interpreted cautiously, as the measure of perceived behavioral control combined self-efficacy-oriented and controllability-oriented elements within a single construct. The result therefore suggests that the application of TPB to disability-related digital health contexts may require greater conceptual and methodological attention to the multidimensional nature of perceived control.

Overall, the integrated Technology Acceptance Model–Theory of Planned Behavior framework provided a useful, although incomplete, explanation of intention to adopt digital health technologies among people with disabilities. These findings highlight the importance of perceived benefit, positive evaluation, social support, accessible implementation conditions, and disability-specific health promotion needs. Future research should employ more differentiated measures of perceived control, include more diverse disability groups and cultural contexts, incorporate additional variables such as accessibility and digital literacy, and examine actual use behavior in addition to intention.

## Figures and Tables

**Table 1:** Demographic, Disability-Related, and Digital Health Technology Experience Characteristics of Participants.

Variable	Category	n	%
Gender	Male	210	58.0
	Female	152	42.0
Age (years)	20–39	98	27.1
	40–59	178	49.2
	60 or older	86	23.7
Type of disability	Physical impairment	231	63.8
	Hearing impairment	82	22.7
	Visual impairment	49	13.5
Severity of disability	Mild	147	40.6
	Moderate	167	46.1
	Severe	48	13.3
Digital health technology experience	Yes	228	62.9
	No	134	37.1

Note. n = number of participants; % = percentage of the total sample.

**Table 2:** Measurement Items.

Factor	Code	Item	Cronbach’s α
PEOU	PEOU1	Learning to use digital health technologies for physical activity would be easy for me.	0.824
	PEOU2	I would be able to use digital health technologies for physical activity without much difficulty.	
	PEOU3	Using digital health technologies for physical activity would not be complicated for me.	
	PEOU4	I could become skillful at using digital health technologies for physical activity without much effort.	
PU	PU1	Using digital health technologies for physical activity would improve my exercise management.	0.883
	PU2	Digital health technologies for physical activity would improve the effectiveness of my health management.	
	PU3	Using digital health technologies would help me maintain regular participation in physical activity.	
	PU4	Digital health technologies for physical activity would be useful in enhancing my exercise participation.	
ATT	ATT1	Using digital health technologies for physical activity is positive.	0.866
	ATT2	Using digital health technologies for physical activity is desirable.	
	ATT3	I have a favorable attitude toward using digital health technologies for physical activity.	
	ATT4	Managing physical activity through digital health technologies is valuable to me.	
	ATT5	Using digital health technologies for physical activity is a good choice.	
SN	SN1	People close to me would support my use of digital health technologies for physical activity.	0.749
	SN2	People who are important to me would want me to use digital health technologies to manage my health and physical activity.	
	SN3	The opinions of instructors, professionals, or people around me would influence my use of digital health technologies for physical activity.	
PBC	PBC1	If I wanted to, I would be able to use digital health technologies for physical activity.	0.725
	PBC2	I am confident that I can use digital health technologies for physical activity on my own.	
	PBC3	My use of digital health technologies for physical activity depends largely on my willingness and circumstances.	
INT	INT1	I intend to use digital health technologies for physical activity in the future.	0.922
	INT2	If given the opportunity, I would be willing to use digital health technologies for physical activity.	
	INT3	I am likely to use digital health technologies to manage my physical activity in the future.	
	INT4	I plan to continue using digital health technologies for physical activity.	

Note. All items were measured on a five-point Likert scale ranging from 1 = strongly disagree to 5 = strongly agree. PEOU = perceived ease of use; PU = perceived usefulness; ATT = attitude toward use; SN = subjective norm; PBC = perceived behavioral control; INT = intention to use. The PBC measure reflects a mixed operationalization: PBC1 and PBC2 are primarily self-efficacy-oriented, whereas PBC3 also captures circumstance-related controllability.

**Table 3:** Confirmatory Factor Analysis Results.

Construct	Item	Factor Loading	CR	AVE	√AVE
Perceived ease of use	PEOU1	0.855	0.840	0.579	0.761
	PEOU2	0.894			
	PEOU3	0.742			
	PEOU4	0.507			
Perceived usefulness	PU1	0.776	0.875	0.638	0.799
	PU2	0.833			
	PU3	0.848			
	PU4	0.790			
Attitude	ATT1	0.717	0.876	0.589	0.767
	ATT2	0.670			
	ATT3	0.762			
	ATT4	0.827			
	ATT5	0.806			
Subjective norm	SN1	0.843	0.820	0.613	0.783
	SN2	0.884			
	SN3	0.572			
Perceived behavioral control	PBC1	0.822	0.749	0.510	0.714
	PBC2	0.588			
	PBC3	0.579			
Intention to use	INT1	0.736	0.900	0.698	0.835
	INT2	0.768			
	INT3	0.936			
	INT4	0.898			

Note. CR = Composite Reliability; AVE = Average Variance Extracted. All factor loadings are significant at *p* < 0.001.

**Table 4:** Construct Correlations and Discriminant Validity.

Construct	PEOU	PU	ATT	SN	PBC	INT
PEOU	0.761					
PU	0.677	0.799				
ATT	0.687	0.792	0.767			
SN	0.716	0.631	0.713	0.783		
PBC	0.552	0.509	0.604	0.567	0.714	
INT	0.288	0.330	0.345	0.294	0.052	0.835

Note. Diagonal values represent the square root of average variance extracted (AVE) for each construct. Off-diagonal values represent inter-construct correlations. PEOU = perceived ease of use; PU = perceived usefulness; ATT = attitude; SN = subjective norm; PBC = perceived behavioral control; INT = intention to use.

**Table 5:** Model Comparison: Model 1 vs. Model 2.

Path		Model 1 β	Model 1 *p*	Model 2 β	Model 2 *p*	Note
Model Fit Indices	χ^2^/df	2.545		2.528		
	CFI	0.931		0.931		
	TLI	0.920		0.921		
	RMSEA	0.065		0.065		
	AIC	670.705		668.247		← Lower
Structural Paths	H1: PEOU → PU	0.677	<0.001	0.677	<0.001	Supported
	H2: PEOU → ATT	0.229	<0.001	0.232	<0.001	Supported
	H3: PU → ATT	0.671	<0.001	0.672	<0.001	Supported
	H4: PEOU → PBC	0.355	<0.001	0.350	<0.001	Supported
	H5: PU → PBC	0.297	<0.001	0.298	<0.001	Supported
	H6: PU → INT	0.055	0.662	—	—	Excluded
	H7: PEOU → INT	0.109	0.353	—	—	Excluded
	H8: ATT → INT	0.325	0.013	0.410	<0.001	Supported
	H9: SN → INT	0.088	0.315	0.139	0.042	Significant in Model 2 only
	H10: PBC → INT	−0.227	0.007	−0.204	0.008	Not Supported †

Note. Model 1 includes the direct paths from perceived usefulness (PU) and perceived ease of use (PEOU) to intention, whereas Model 2 excludes these paths. β = standardized regression coefficient. † Significant but in the direction opposite to the hypothesis. AIC = Akaike Information Criterion; lower values indicate better model parsimony. The arrow symbol (→) indicates the hypothesized direction of the structural path. “← Lower” indicates the model with the lower AIC value.

**Table 6:** Final Structural Model Results (Model 2).

Hyp.	Path	β	C.R.	*p*	Result
H1	PEOU → PU	0.677	11.024	<0.001	Supported
H2	PEOU → ATT	0.232	3.727	<0.001	Supported
H3	PU → ATT	0.672	9.445	<0.001	Supported
H4	PEOU → PBC	0.350	4.075	<0.001	Supported
H5	PU → PBC	0.298	3.538	<0.001	Supported
H6	PU → INT	—	—	—	Excluded
H7	PEOU → INT	—	—	—	Excluded
H8	ATT → INT	0.410	5.242	<0.001	Supported
H9	SN → INT	0.139	2.034	0.042	Supported
H10	PBC → INT	−0.204	−2.669	0.008	Not Supported †

Note. β = standardized regression coefficient; C.R. = critical ratio (t-value). † Significant but in the direction opposite to the hypothesis. H6 and H7 were excluded from Model 2 due to non-significance in Model 1. The arrow symbol (→) indicates the direction of the structural or indirect path.

**Table 7:** Bootstrapping Results for Indirect Effects (Model 2).

Indirect Path	Std. β	BC *p*	95% CI	Interpretation
PEOU → INT (Total)	0.169	0.004	[0.042, 0.284]	Significant
PEOU → ATT → INT	0.095	-	-	Positive via ATT
PEOU → PU → ATT → INT	0.186	-	-	Sequential via PU → ATT
PEOU → PBC → INT	−0.071	-	-	Negative via PBC
PEOU → PU → PBC → INT	−0.041	-	-	Negative via PU → PBC
PU → INT (Total)	0.214	<0.001	[0.068, 0.207]	Significant
PU → ATT → INT	0.276	—	—	Positive via ATT
PU → PBC → INT	−0.061	—	—	Negative via PBC

Note. The arrow symbol (→) indicates the direction of the structural or indirect path.

**Table 8:** Comparison of the Present Integrated Framework with Recent Digital Health Technology Acceptance Studies.

Study	Population/ Context	Technology Context	Framework/ Focus	Key Contribution	Relevance to the Present Study
Kim et al. [7]	People with disabilities	Digital health technologies	TAM	Examined intention to use digital health technologies among people with disabilities	Provides the most directly relevant empirical basis for disability-related digital health acceptance
Ko et al. [19]	People With disabilities	Telehealth	Scoping review	Identified accessibility, usability, and service support as important issues in telehealth use	Supports the need to consider disability-specific barriers and support conditions
Liu et al. [20]	Sensor-based monitoring context	Structural health monitoring using ultrasonic guided waves	Long-term technical reliability	Proposed a method for long-term temperature compensation and system reliability in sensor-based monitoring	Supports the need to consider technical reliability and robustness in wearable and sensor-based digital health monitoring systems
Löchner et al. [21]	Digital mental health context	Digital interventions	Overview/future perspectives	Emphasized engagement, sustainability, and future directions for digital interventions	Supports the inclusion of contextual fit, user engagement, and long-term sustainability in future models
Kaló et al. [22]	Investigational Digital technologies	Digital mental health technologies	Early-stage assessment	Highlighted perceived value, feasibility, user relevance, and contextual fit before implementation	Supports the practical implication that perceived usefulness alone is insufficient without implementation feasibility
Westheimer et al. [23]	Mental health professionals/ end users	Digital transformation in healthcare	Implementation model	Emphasized end-user support, organizational readiness, and implementation processes	Supports the need for social, organizational, and technical support in digital health adoption
Present study	People with physical, visual, or hearing impairments participating in community-based sports programs	Digital health technologies for physical activity	Integrated TAM–TPB	Tested technology-specific beliefs and social–cognitive determinants in one SEM framework	Extends prior work by applying an integrated model to physical activity among people with disabilities

## Data Availability

The data presented in this study are not publicly available due to privacy and ethical restrictions related to sensitive information from participants. However, the data may be made available from the corresponding author upon reasonable request and with permission from the Institutional Review Board.
